# Acceptance of Vaginal Birth After Caesarean Section Trial in Shree Birendra Hospital, Kathmandu, Nepal: A Descriptive Cross-sectional Study

**DOI:** 10.31729/jnma.5781

**Published:** 2021-01-31

**Authors:** Ratna Adhikari Khatri, Arju Chand, Manish Thapa, Sumana Thapa, Shailaja Khadka

**Affiliations:** 1 Department of Obstetrics and Gynecology, Nepalese Army Institute of Health Sciences, Sanobharyang, Kathmandu, Nepal; 2 Department of Radiology, Nepalese Army Institute of Health Sciences, Sanobharyang, Kathmandu, Nepal

**Keywords:** *antenatal care*, *intrapartum*, *vaginal birth after caesarean section*

## Abstract

**Introduction:**

The rate of primary cesarean section is on the rising trend. Vaginal birth after cesarean section can be an alternative to reduce cesarean section worldwide. Antenatal examination and intrapartum monitoring are the most important factors for a vaginal birth after a cesarean section. This study aims to determine the acceptance of vaginal birth after cesarean section trial in a tertiary care hospital in Nepal.

**Methods:**

This is a descriptive cross-sectional study carried out in Shree Birendra Hospital, Kathmandu, Nepal, from March 2019 to March 2020. All pregnant women with a previous history of cesarean section meeting Royal College of Obstetrics and Gynecology criteria were included. A trial of labor was conducted on the patients who accepted vaginal birth after cesarean section.

**Results:**

A total of 85 cases with previous lower section cesarean section were included in the study. Out of which, 75 (88.2%) refused vaginal birth after cesarean section, and only 10 cases (11.8%) accepted to undergo a trial of labor. Five women (50%) had a successful vaginal birth. Complications were less among the vaginal birth after cesarean section group than the repeat cesarean section group. There was no maternal and neonatal mortality.

**Conclusions:**

The acceptance of vaginal birth after cesarean section is very low in this study. No complications were observed among vaginal birth after cesarean section in our study.

## INTRODUCTION

Vaginal birth after cesarean section (VBAC) trial is an alternative to repeated Caesarean sections (CS). It peaked during the mid-1990s, along with a lower rate of CS. However, the evidence is inconsistent, and the effect on VBAC is unclear. This decline has been a response to new evidence on VBAC's risks and clinician's fear of professional liability.^1,2^ In 1916, Cragin popularized the dictum “once a cesarean section, always a cesarean section.” That was the era of classical CS.^3,4,5^

The dictum now is “once a cesarean section, always an institutional delivery in a well-equipped hospital.”

The reasons that led to the reversal of the old dictum are based on the scar integrity, fetal well-being, and improved emergency CS facilities.^4,5^

The data on the success and failure of the VBAC in Nepal is inadequate. This study aimed to determine the acceptance of a Vaginal Birth Trial after Cesarean Section Trial in a tertiary care hospital of the Nepalese army.

## METHODS

This was a descriptive cross-sectional study carried out at Shree Birendra Hospital, Kathmandu, Nepal, from March 2019 to March 2020. The study was approved and vetted by the Institutional Review Committee in March 2019. All pregnant women with a previous history of lower segment Caesarean section (LSCS) to the antenatal Outpatient Department, meeting the Royal College of Obstetrics and Gynecology criteria for VBAC, were included in the study. Women with a history of more than one cesarean section, cephalopelvic disproportion, severe preeclampsia, eclampsia, and antepartum hemorrhage, multiple pregnancies, malpresentation, malposition, short spacing less than 18 months, and with other medical histories such as moderate or severe anemia, severe hypertension, diabetes mellitus, renal disease, and heart disease were excluded.

The sample size was calculated using the following formula:

$$\begin{array}{ll} \text{n} & ={\text{Z}}^{\text{2}}\text{pq}/{\text{e}}^{\text{2}}\\ & ={\left(1.96\right)}^{\text{2}}\times 0.11\times 0.89/{(\text{0.07)}}^{\text{2}}\\ & =77 \end{array}$$

Where,

p is the prevalence from reference study^3^,
i.e. 11.0%.
q is the compliment of p, i.e., q=100-p
d is the allowable error, 7%

Z is the standard normal variate, which is 1.96 for a 95% confidence interval.

Hence, the calculated sample size for this study is 77. However, we included all the pregnant women with a previous history of LSCS during the study period.

The data was collected via an interview using a pretested proforma. The proforma consisted of demographic variables such as age, occupation, and educational status and obstetric history such as gravida, gestational age, history of previous pregnancies, cause of previous CS, intraoperative and postoperative details from the documents if they possess or if they could recall. Detailed physical and obstetric examination along with fundal height, lie, presentation, position, scar tenderness, and fetal heart rate were recorded. After evaluation, the patients were thoroughly counseled regarding the potential benefits and risks of undergoing a trial of labor and gave her a choice to choose the mode of delivery.

A trial of labor was conducted, and progress was partographically monitored. Non-progressing labor was identified as per the definition of Freidman for labor complications. Evaluation of fetal head descent by abdominal examination, cervical dilatation, and effacement, progressive increase in frequency, duration, and intensity of uterine contraction noted. Patients in spontaneous labor were closely monitored for vital signs, fetal cardiac activity, lower abdominal tenderness, fetal distress, vaginal bleeding, and urine retention. Signs and symptoms of scar dehiscence or rupture were monitored judiciously. Depending upon the clinical evaluation decision regarding the use of oxytocin and amniotomy was taken. If labor progress was satisfactory, the trial of labor continued and allowed to deliver vaginally with an episiotomy or vacuum if needed. Facilities for emergency C-sections were made available, and postnatal patients with normal delivery were observed for 48 hours for vital signs, postpartum hemorrhage, or any other complications. Any sign of danger to the mother or fetus during labor led to an emergency C-section. The percentage of vaginal delivery determined the success of VBAC. Neonatal outcome was analyzed concerning baby weight and APGAR score and NICU admission in the context of VBAC and repeat CS.

The data were entered in Microsoft Excel 2007 and analyzed using Statistical Package for Social Sciences (SPSS) Version 17. Descriptive analysis was applied to show the categorical data in terms of frequencies and percentages while continuous data as means. Informed verbal consent was obtained from all the patients before the data collection.

## RESULTS

Out of 108 total previous CS cases, 23 were excluded from the study. The excluded were 5 cases of short spacing 18 months and below, 5 cases with a high-risk pregnancy, 4 cases of previous 2 LSCS, 4 cases in labor with scar tenderness, 3 cases of a big baby more than 3.5kg, and 1 case of premature rupture of membrane (PROM) with big fibroid and 1 case with multiple pregnancies. Thus, the remaining 85 (78.7%) women were included in the study group as a potential candidate for a VBAC. Out of 85 cases, only 10 (11.76%) cases agreed to the VBAC trial, and 75 (88.23%) cases refused and underwent elective CS.

The commonest age group that agreed to the VBAC trial was 25-30 years. The success rate of VBAC was found to be 50% (Table 1). Among the participants who accepted the VBAC trial, a majority 5 (50%) had Caesarian section due to Fetal Distress (Table 2).

**Table 1:** Distribution of participants who agreed to VBAC by age and outcome of VBAC.

Age (in years)	n (%)	Successful VBAC n (%)	Failed VBAC n (%)
20-24	1 (10)	1 (100)	0 (0)
25-30	5 (50)	3 (60)	2 (40)
31-34	2 (20)	1 (50)	1 (50)
35-37	2 (20)	0 (0)	2 (100)
Total	10 (100)	5 (50)	5 (50)

**Table 2:** Indications of previous LSCS among participants who accepted VBAC and its outcome.

Indications of Previous CS	n (%)	Successful VBAC n (%)	Failed VBAC n (%)
PROM with Oligohydrominous	1 (10)	0 (0)	1 (100)
Fetal distress	5 (50)	4 (80)	1 (20)
Crossed Expected date of delivery	1 (10)	0 (0)	1 (100)
Cephalopelvic disproportion	1 (10)	1 (100)	0 (0)
Failed Induction	1 (10)	0 (0)	1 (100)
Transverse lie	1 (10)	0 (0)	1 (100)
Total	10	5	5

Among 5 patients with failed VBAC, 4 underwent emergency LSCS (2 for failed induction and 2 for scar tenderness), and 1 underwent elective LSCS for crossing EDD (Table 3).

**Table 3:** Indications of cesarean section in failed VBAC.

Patient Characteristics	Outcome	n (%)
Failed Induction	Emergency LSCS	2 (40)
Crossed EDD	Elective LSCS	1 (20)
Scar Tenderness	Emergency LSCS	2 (40)
Total		5

There were no complications encountered in the successful VBAC group. We observed one case of wound infection in a failed VBAC who underwent emergency CS for failed induction with oxytocin. Among those who underwent repeat CS (rejected VBAC), 17 (22.6%) cases had wound infection, 3 (4%) cases had a postpartum hemorrhage, and 8% had puerperal pyrexia and, 11 cases had scar tenderness with dehiscence.

2 cases in successful VBAC needed NICU admission (Table 4). There was no neonatal mortality among the study population.

**Table 4:** Neonatal Outcomes in VBAC and repeat CS groups.

Neonatal outcome	Successful VBAC n (%)	Failed VBAC n (%)
Mean Neonatal Weight in grams	3100	2950
Apgar score ≤ 6 in 1 min	2 (40)	4 (80)
Apgar score >6 in 1 min	3 (60)	1 (20)
NICU admission	2 (40)	0 (0)
Total	7	5

## DISCUSSION

CS is one of the most performed major surgical procedure.^6^ Increase in CS rate during the last three decades has been a cause for concern worldwide. The rates of CS varies across countries from 10% to 40%.^7–9^ The reasons women adopt cesarean delivery include medical considerations such as complications during pregnancy or labor and delivery processes, and non-medical causes such as fear of pain, late marriages, patient's demand, or more predictable delivery date and time. It is particularly common in Chinese and Asian culture to have babies delivered at certain “auspicious” points in time. Many Chinese believe that a person's positive destiny is crucially determined by the time and date of that person's birth.^6^ On analyzing, these are some of the factors which are the causes of increasing CS rate in our institution especially on demand CS.

Although attempts at the trial of labor after CS have become accepted practice, the rate of attempted and successful VBAC has decreased during the past 10 years in the developed world. The number of patients attempting VBAC has drifted down in the developing world from 20% to 10% during 2002-2005.^10^ In the USA, the overall rate of VBAC (i.e., successful VBAC/ all women with a previous CS) decreased from 24% in 1996 to 8% in 2010.^10^ A qualitative study from the USA suggest that fear of litigation is a further reason why providers are highly selective in choosing candidates for VBAC.^11^ In our study, the VBAC rate is found to be 11.7%, which is comparable to studies done in a tertiary care center in Eastern Nepal (11%) and Pakistan (10.4%).^3,12^ These rates are much lower than many other studies conducted in developed countries. The VBAC rate depends upon a combination of factors, including the type of healthcare system, patient preferences, and the extent to which national clinical guidelines recommend VBAC.

Many cases refused for the trial of VBAC in this study. Attempts at vaginal delivery were abandoned, at every moment, when there was even a bit of suspicion of scar dehiscence and also to avoid neonatal morbidity and lastly, patient's unwillingness after that, for a trial of labor. A similar finding was obtained in a study conducted at Tribhuvan University teaching hospital, Maharajgunj, Kathmandu by Pooja et al. as VBAC remained at 0.15% to 0.7%.^13^ The acceptance for the trial of labor in this study group was very low to come to any conclusion, similar to the study conducted by Knight et al. where, among 50,000 women in England, just over a one-half attempt to give birth vaginally following VBAC. The mother's choice of delivery mode is the most important single factor in offering a trial of labor. In the context of less acceptance for VBAC, studies conducted by clinicians in Germany and Ireland suggested that giving information early in the pregnancy helps build a woman's confidence that she can achieve a VBAC.^14^ Likewise, in our study thorough counseling of the patients about the VBAC did work, but it again raised the issue of continuity of care by the same obstetrician and midwives all the time. In teaching institutions like ours, it is very difficult to maintain the continuity of care by the same obstetrician and midwives all the time for one patient.

Less acceptance of the patients for a trial of labor may also be due to the fear of labor pains, and another most concerning issue to both patients as well as the doctor is the uterine rupture. In this study, we could observe 11 cases of scar dehiscence who had undergone repeat CS, which again plays a vital role in lowering the rate of VBAC due to fear of neonatal and maternal morbidity and litigation to the doctors. This study is unlike to study done in Pakistan by Qazi et al., where only one-third of total previous CS cases had VBAC due to a lack of sophisticated monitoring devises in their set up, which was coinciding with the findings by Yousaf et al. and Elkhousy et al.^8,10,15^

In this study, a high success rate of 80% for VBAC was achieved for cases of non-recurrent causes of primary CS like fetal distress, similar to other studies reporting a 60 to 80% success rate.^7^

During 1996-2000, the VBAC rate in California decreased from 23% in 1996 to 15% in 2000, a decline by 35% is reported after maternal race/ethnicity, age, insurance status, and education were stratified, a consistent downward trend in VBAC rates was observed for all populations. It has been reported that college graduates had the highest VBAC rates, and women with less than a high school education had the lowest rates; declines in VBAC rates were similar among women of all education levels.^16^ Similar finding was found in the present study where acceptance for the VBAC was more from the college graduates and declination were observed more among the highly educated group.

Studies have shown that women with Health Maintenance Organization coverage had the highest VBAC rates, and women with MediCal/Medicaid had the lowest rates.^16^ Likewise, the present study location is a welfare hospital run by the Nepalese army, where the medical expenses of the dependents of the army personal are free, so this factor has also played a major role for patients not accepting to undergo a trial of labor as there is no financial burden on the patients. This may be why the majority 89% of the patients refused to accept VBAC and ultimately landed up in CS. This is a significant financial burden on the institution.

A similar study in Taiwan reported that women from wealthier families are less likely to undertake VBAC. In contrast, older women and women with higher fertility are more likely to undertake VBAC.^6^ All our study population belongs to the army and hence can maintain the standard of living, which may be the reason for reduced acceptance of VBAC in our institution.

Contrary to other studies^17^, the acceptance for labor trial was more from the elderly age group in this particular study. It was observed that the success of VBAC was more with patients who had not crossed 40 weeks (i.e., between 38-39 weeks) of the period of gestation (POG). Failed induction and scar dehiscence were observed more among cases who had crossed 40 weeks POG. This was a vital clinical observation for the decision of VBAC in the future, which is also supported by studies conducted in China and Thailand.^14,17,18^ In the delivery process, Bishop score ≥5 had successful VBAC, whereas women with rupture of membrane and using oxytocin augmentation were significantly less likely to achieve the success of VBAC.^17^ A cohort study conducted in Thailand also showed that late gestational age was significantly associated with a higher failure rate.

This study further strengthens the observations made by previous studies that the success of VBAC is more in cases where an emergency LSCS was performed in the previous pregnancy. All successful VBAC had emergency CS previously. So, we recommend that a fair trial of VBAC be given to all the patients who fulfill RCOG/ACOG criteria for VBAC with a single previous CS. Spontaneous progress of labor was a favoring point for the successful VBAC in the present study. We observed that 5 patients who had successful VBAC all had spontaneous onset of labor, which gave 100% success for VBAC, whereas cases induced with oxytocin followed by rupture of the membrane had failed induction and scar dehiscence. Contrary to this, among the unaccepted cohort, the patients who had vaginal delivery in a previous pregnancy, none had accepted for the trial of labor may be due to fear of labor pains and concern for neonatal morbidity.

VBAC is associated with a short period of hospitalization, less blood loss and fewer transfusions, fewer infections, and fewer thromboembolic events than a cesarean delivery. Several reports have indicated that the absolute risk of uterine rupture attributable to a trial of labor is about 1 per 1000. A successful VBAC has fewer complications than an elective repeat CS. There was no case of uterine rupture, lesser transfusion, and infections in the VBAC group in the present study. The hospitalization for repeat CS is much more than the successful VBAC due to wound infection, increase blood loss during surgery, leading to blood transfusions. The average duration of hospital stay for the VBAC group was 4.6 days, whereas it was 8.4 days in the repeat CS group.^4^ Benson et al. also found that a shorter hospital stay in a VBAC delivery has a positive impact on the woman's psychology and decreases the total cost of hospitalization.^19^ Similar observations were made by another study.^18^

In our study, the neonatal morbidity was more (80%) in failed VBAC with APGAR < 6, whereas in elective repeat CS cases, it was only 36%. NICU admission was 20% in the failed VBAC group, whereas 12% in the elective repeat CS cohort. The scar dehiscence in the failed VBAC group was 20%, whereas, in the elective repeat CS cases, it was 12%. These findings were similar to the study conducted by Young et al., where higher relative rates of severe morbidity and mortality in mothers and infants were observed in attempted VBAC.^20^ Neonatal morbidity was due to failed induction and prolonged labor with a cord around the neck. All three babies had developed birth asphyxia for a transient period. Maternal and neonatal morbidities were higher among repeat CS and failed VBAC groups than those with successful VBAC, similar to the study's findings in India.^4^ However, our study did not reveal any perinatal mortality or maternal mortality. Contrary to our finding, Jha et al. reported infants born after successful VBAC 36% had the lowest NICU admission rates and the lowest resuscitation needs; those born otherwise 13% had the highest resuscitation needs.^21^ Qazi et al. also concluded that the failed trial of labor is at increased risk of jeopardized fetal conditions, and operative interference should be made in time if complications like fetal or maternal distress come into the picture.^10^ The clinician must respect the patient's autonomy and decision-making capabilities while considering the route of delivery after counseling her about all maternal and fetal risks.^10,22^

There are some limitations to the study. First, a very high percentage of women, 88.2%, denied a trial of labor. The sample size is very small to conclude the prevalence of successful VBAC and compare maternal and neonatal adverse events. As this study is done in one institution, hence cannot be generalized.

## CONCLUSIONS

The acceptance of the VBAC trial in our study is very low. Stringent selection criteria and meticulous intrapartum monitoring often lead to successful VBAC. It helps in the reduction rate of CS and thereby reducing maternal morbidity due to repeat CS. The majority of previous CS cases done for non-recurrent indications can be delivered safely by the vaginal route, without any major complication to the mother and the newborn, in an institution well-equipped for emergency CS.

Proper counseling for a trial of labor and evaluation of the case is a key method of reducing the CS rate. This research encourages Obstetrics to encourage VBAC in the properly screened antenatal patients and decrease CS rate. This decreases the financial burden to the organization and helps decrease healthcare expenditure and avoid over-crowding in the tertiary care hospital like ours.
